# Nanoscale mapping of residual stresses in Al 2024 alloys using correlative and multimodal scanning transmission electron microscopy

**DOI:** 10.1016/j.heliyon.2024.e30280

**Published:** 2024-04-26

**Authors:** Mohamed E. Daoud, Inas Taha, Mohamed Helal, H. Kamoutsi, G.N. Haidemenopoulos, Kamran A. Khan, Dalaver H. Anjum

**Affiliations:** a Department of Physics, Khalifa University, P.O. Box 127788, Abu Dhabi, United Arab Emirates; b Core Research Laboratories, Khalifa University, P.O. Box 127788, Abu Dhabi, United Arab Emirates; c Laboratory of Materials, Department of Mechanical Engineering, University of Thessaly, Pedion Areos, 38334, Volos, Greece; d Aerospace Engineering Department, Khalifa University, P.O. Box 127788, Abu Dhabi, United Arab Emirates

**Keywords:** Residual stresses, Scanning transmission electron microscopy (STEM), Electron energy loss spectroscopy (EELS), Four-dimensional transmission electron microscopy (4D STEM), Young's modulus, Al 2024 alloy

## Abstract

A methodology for the mapping of residual stresses in metal alloys has been developed by analyzing an isotropic and homogeneous Al2024 alloy with scanning transmission electron microscopy (STEM), combined with diffraction (4DSTEM) and electron energy loss spectroscopy (STEM-EELS) techniques of TEM. The investigations on the alloy's microstructure and elemental distributions were also carried out with conventional dark-field STEM (DFSTEM) and X-ray energy dispersive (EDS) techniques, respectively. Using the STEM-EELS technique, the Young's modulus (*Y*_*M*_) is mapped in the (001) plane of the Al alloy in the same regions where the residual strain maps are generated in [$\bar{1}$ 00] and [010] directions by using 4DSTEM technique. The $Y_{M}$ vs. residual strain plot for the Al 2024 alloy revealed that the value of $Y_{M}$ decreased by about ∼ 7 % after the tensile residual strain reached 0.02 %. Whereas such a decrease in $Y_{M}$ happens after the compressively residual strain reaches −0.015 %. The residual stress maps were also obtained in accordance with the Hooke's law i.e., by multiplying $Y_{M}$ map with the corresponding residual strain maps.

## 1 Introduction

Residual stresses, also referred to as trapped or locked stresses, arise during various manufacturing processes of engineering components of metal or metal alloys [1,2]. An intriguing characteristic of these stresses is their existence in solid materials without the presence of external mechanical or thermal loads. When the raw materials are manufactured into the final product, there are processes along the way, such as heat treating, forging, welding, machining, and surface treatments, which generate residual stresses to the final product [3,4]. There are three main ways in which these processes can create residual stress: thermal processes, such as solidification of a material in a cooling casting; machining processes, such as plastic deformation of a material during machining; and phase changes, such as precipitation or phase transformation [5]. Residual stress can be tensile or compressive and depending on the application, both can be an advantage or disadvantage. For example, compressive stress can increase fatigue life and delay crack propagation [3,5]. These stresses are typically categorized by their operational length scale [1,3]: Type I stresses, or macro residual stresses, manifest at the component's overall geometry scale. Type II, often termed intergranular or micro stresses, operates at the grain level. Lastly, Type III stresses function at the atomic scale, resulting from atomic discrepancies like vacancies or the introduction of substitutional atoms. Residual stresses can be measured using destructive techniques such as hole drilling method and milling method, or non-destructive such as X-ray diffraction, neutron diffraction, and ultrasonic method [[6], [7], [8]]. In most measurement techniques of residual stresses, Young's modulus is assumed to be constant [9,10]. For example, Diffraction-based (X-ray and neutron) methods that are used to determine residual stresses at the microscale assume the residual stress is related to the residual strain via a constant value of the Young's modulus [7,8]. Zhao et al. [7], used the X-ray diffraction method also known as the $\sin^{2}\;\psi$ method to determine residual stress in a selected region of dual-phase steel of DP600 sample after performing a uniaxial tensile test. This method utilizes the interplanar spacing of crystallographic planes as a gauge for measuring residual stress levels in materials. The residual stress was calculated using the following equation:

(1) $$\sigma_{\psi }=\left[Y_{m}/2\left(1+v\right)\right]\;\cot\;\theta_{ο}\left[\pi /180\right]\left[\partial 2\theta_{\psi }/\partial \sin^{2}\;\psi \right]$$

where $\sigma_{\psi }$ is the residual stress, $Y_{m}$ is the Young's modulus, *ν* is the Poisson's ratio of the material, $\theta_{ο}$ diffraction angle under the condition of a no-stress state, and $\theta_{\psi }$ diffraction angle under the condition of the stress state. The neutron diffraction method was utilized by Sutton et al. [8], allowing us to determine the residual stress in Al 2024-T3 alloy both in normal and shear directions. The residual stress tensor for these experiments can be calculated using the following equation:

(2) $$\sigma_{ij}=\left[Y_{m}/\left(1+v\right)\right]\;\varepsilon_{ij}+\left[vY_{m}/\left(1+v\right)\left(1-2v\right)\right]\delta_{ij}\varepsilon_{kk}$$

where $\sigma_{ij}$ is the normal sand shear components of the residual stress tensor, $Y_{m}$ is the Young's modulus, *ν* is the Poisson's ratio of the material, $\varepsilon_{ij}$ is the normal and shear components of the strain tensor, $\delta_{ij}$ is the Kronecker's delta function, and *k* is a dummy index summing over all *k* (*k* = 1,2,3). Only few studies have been conducted for the measurement of the residual stresses at the nanoscale compared to the microscale and macroscale, even though the presence of Type II and Type III residual stresses (RS) can sometimes cause the total RS value to be up to twice as large as the Type I residual stresses (macroscopic RS) [11], which highlights the importance of accurately assessing RS not just on a larger scale, but also at the micro and nano levels, where fatigue failures often begin. Techniques such as Raman spectroscopy [11], Nanoindentation [12], and Focused Ion Beam (FIB) milling [13], also rely on the assumption that the Young's modulus remains constant when they calculate residual stresses.

In the above-mentioned micro and nanoscale techniques, $Y_{m}$ is taken as a constant to calculate the residual stresses. Furthermore, nanoscale residual stresses have not been investigated enough in the literature compared to the microscale and macroscale residual stresses.

In this paper, the residual stresses are calculated from the residual strains determined by using the four-dimensional transmission electron microscopy (4DSTEM) technique and $Y_{m}$ determined with the scanning transmission electron microscopy combined with the electron energy loss spectroscopy (STEM-EELS) method at the same region and spatial resolution where the strains have been determined. Both methods have been applied in a correlative manner and enable determining these quantities at the nanoscale.

## 2 Materials and methods

The material utilized for this study was Al 2024-T351 alloy, provided by Airbus, with a thickness of 2 mm. The chemical composition of the investigated Al2024 alloy is Al-4.35Cu-1.5Mg-0.64Mn-0.5Si-0.5Fe (wt.%). Three specimens of 20 × 20 × 2 mm^3^ were cut from the sheet. The specimens were solution treated at 495 °C for 30 min and water quenched. Subsequently, aging was performed at 170 °C for 9 h, which corresponds to the underaged condition according to Ref. [14]. In this way, the samples presumably possessed a minimum level of residual stresses. The details on the application of T351 temper on the samples can be found elsewhere [15].

For the analysis of residual stresses in samples, the TEM lamellae were prepared using a Helios Nano Lab 650 FIB/SEM dual-beam system from ThermoFisher Scientific. To protect the top surface of the sample, platinum (Pt) layers were deposited on the surface region of interest using electron and ion beam techniques. The samples were then gradually thinned down to a relative thickness of 100 nm by progressively reducing the ion beam energies in the FIB until reaching 2 keV. In this way, the gallium (Ga) ion implantation and the amorphization of the lamella surfaces were minimized. The prepared specimens were analyzed using a double aberration-corrected TEM of model Titan ThemisZ, also from ThermoFisher Scientific which was equipped with an energy filter of model GIF Continuum 1069HR with K3 detector. The accelerating voltage of 300 kV was set during the analysis. The microscope was aligned in STEM mode to have a spherical aberration coefficient (Cs) of ∼2 μm to reach the point resolution of about 70 p.m. Moreover, a high-angle annular dark-field (HAADF) detector was utilized to produce conventional dark-field scanning transmission electron microscopy (DFSTEM) images. In addition, X-ray energy dispersive (EDS) datasets were collected using the high-efficiency 4-sector EDS detector known as the SuperX, provided by ThermoFisher Scientific to have a total solid angle of 2.4 steradian. The post-processing of STEM and STEM-EDS datasets was performed using the Velox Software Package of version 2.2.

The $Y_{M}$ maps were generated by acquiring the low-loss EELS datasets in microprobe STEM mode. The pixel size in acquired STEM-EELS datasets was around 2 nm, while EELS datasets were acquired with a dispersion of 0.09 eV per channel. Furthermore, the collection angle (β) for the acquisition of EELS spectra was ∼20 mrad by setting the camera length of 37 mm and GIF entrance aperture of 5 mm size. Additionally, the C2 condenser lens aperture size of 70 μm was used during the data acquisition. The subsequent post-processing of the acquired STEM-EELS spectrum-imaging (SI) datasets in which each pixel contained information on the HAADF intensity as well as bulk plasmon energy (E_p_), which is obtained from the acquired low-loss EELS spectra. The value of *Y*_*M*_ at each pixel was determined from the measured E_p_ by using the following equation [16]:

(3) $$Y_{M}=0.08\;E_{P}^{2.5}$$

The determination of E_p_ at each pixel of STEM-EELS datasets was carried out using the non-linear least square (NLLS) method available in GMS of version 3.5. It enabled obtaining the E_p_ values at each pixel which were plugged into equation (3) to generate the $Y_{M}$ map for the alloy. Equation (3) holds because it's both sides are proportional to the valence electron density [[16], [17], [18]]. The proportionality constant of 0.08 represents the aggregated numerical value derived from a set of constant physical quantities that are martial dependent.

4DSTEM technique was utilized to record spatially dispersed diffraction patterns in microprobe STEM mode. The diffraction patterns were collected using a K3 direct electron detector in image mode (with binning 4, size 1/4), to gain high speed and high signal-to-noise ratio in the patterns. The acquired datasets were processed with STEMx software in GMS of version 3.5 to the strain maps along x [$\bar{1}$ 00], and y [010] directions when the electron beam was in the [001] direction. The STEMx allowed determine the $u_{x}$ and $u_{y}$ displacement vectors whose derivatives are equal to the strain components when calculated using the following equations [19]:

(4) $${\varepsilon_{\left[\bar{1}00\right]\;}=\varepsilon }_{xx}=\partial u_{x}/\partial x,\varepsilon_{\left[010\right]}=\varepsilon_{yy}=\partial u_{y}/\partial y,\varepsilon_{xy}=\frac{1}{2}\left[\left(\partial u_{x}/\partial x\right)+\left(\partial u_{y}/\partial y\right)\right]$$

Where $\varepsilon_{xx}$ **,** $\varepsilon_{yy}$ are the normal strains and $\varepsilon_{xy}$ is the shear strain component. The residual stress maps can be determined directly from equations (3), (4) using Hooke's Law which is given below:

(5) $$\sigma_{ij}=Y_{M}\;\varepsilon_{ij}$$

The validity of equation (5) demands that both Young's modulus and strain must be determined from the same regions, and therefore, this has been achieved by ensuring the same size of spectrum imaging datasets for the cases of STEM-EELS and 4DSTEM. The residual stress maps were generated in the same region by multiplying the $Y_{M}$ and the residual strain maps per equation (5) using the GMS software.

## 3 Results and discussion

**Fig. 1:**
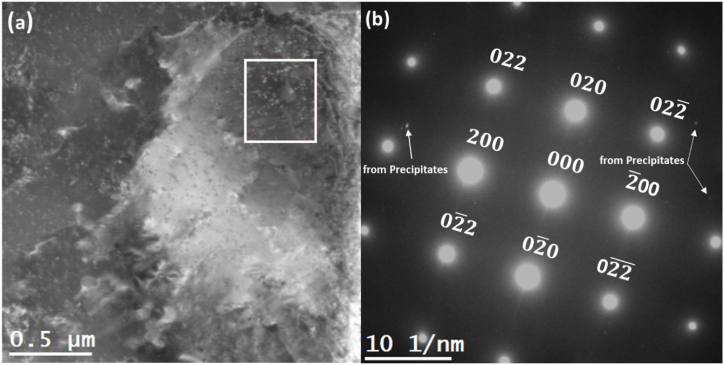
AA2024 microstructure observed using double aberration-corrected STEM (a) A DF-STEM image acquired showcasing dark-field contrast of precipitates along with the Al-matrix. A specific region of interest within this image, indicated by the white box, was chosen for further analysis, from which the SAED pattern was acquired. (b) The SAED pattern was acquired from the region of interest shown by the white box on the DF-STEM image.

**Fig. 2:**
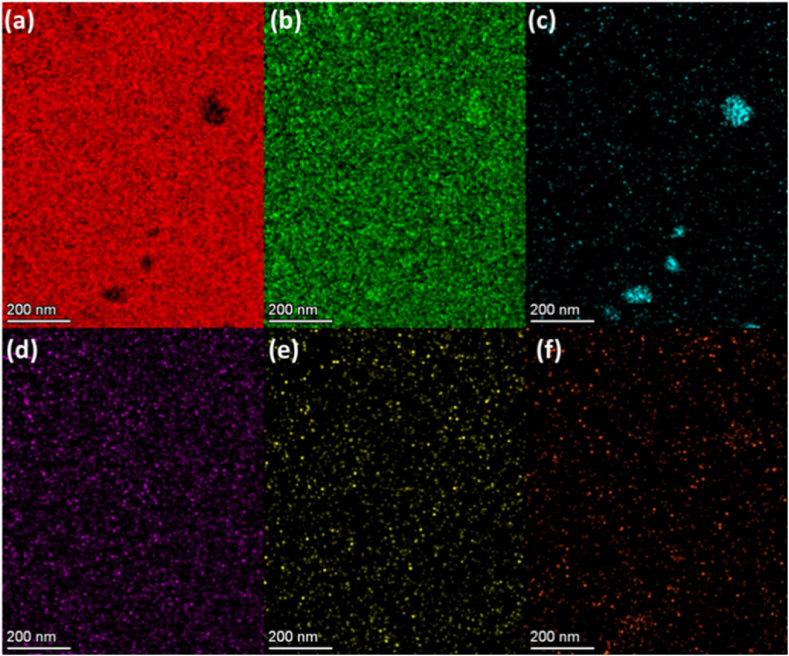
STEM-EDX elemental maps of the region of interest of elements (a) Al, (b) Cu, (c) Mn, (d) Mg, (e) Si, and (f) Fe.

A detailed examination of the microstructure using the DF-STEM technique revealed the microstructure of the alloy. Fig. 1(a) contains a DF-STEM image that shows a conspicuous enrichment of the alloy matrix with precipitates. DF-STEM image in Fig. 1 (a) also illustrates a uniform distribution of the precipitates in the Al matrix. The existence of these dispersed precipitates within the aluminum matrix demonstrates the successful application of the aging treatment, carrying notable implications for understanding the mechanical characteristics of the Al2024 alloy. The precipitates were analyzed by tilting a specific grain of the foil along the [001] _Al_ zone axis. Fig. 1(b) displays a selected area electron diffraction (SAED) pattern obtained from a region enclosed by a rectangular box in Fig. 1 (a). The acquired SAED pattern exhibits the anticipated diffraction spots corresponding to the aluminum matrix. Also, it contains streaks along the [010] direction due to the presence of precipitates. The lattice constant of the Al matrix with FCC crystal structure was found to be a = 0.405 nm, which matches the value obtained in the literature [20]. The diffraction spots from the precipitates are shown beside the diffraction spots from the FCC matrix in Fig. 2(b) as shown below.

**Fig. 3:**
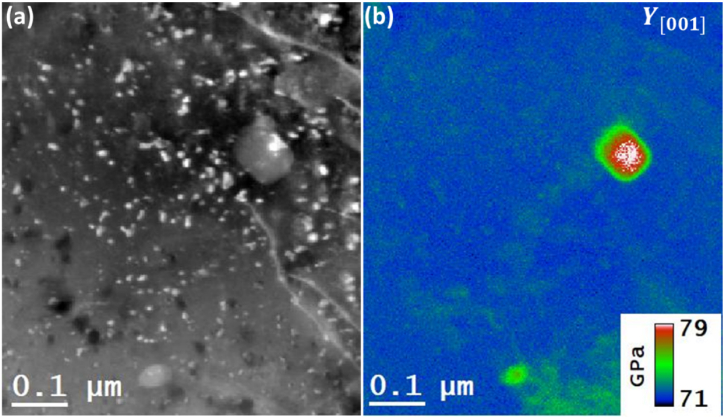
The mapping of Young's modulus ($Y_{M}$) in Al 2024 alloy using STEM-EELS. (a) Region of interest HAADF-STEM image containing precipitates and Al matrix. (b) Mapping of *Y*_*M*_ by applying NLLS on STEM-EELS spectrum image datasets.

The objective of the study was to map the residual stress in the alloy, and the successful completion of this task requires determining the elemental distribution from the same regions because the stress-strain behavior of a material affects elemental composition just as much as under material processing. Changes in any of these factors can affect the material's strength, toughness, and ductility. For example, increasing the amount of manganese-copper locally leads to the formation of dispersoids in aluminium alloys that may increase its strength but generally result in the decrease of its ductility. Fig. 2 contains the sought elemental maps that have been generated using the STEM-EDX spectrum imaging technique. It is to be noted that the presented elemental maps are generated from the same region of interest as enclosed by the rectangular box in Fig. 1(a). The generated elemental maps show that the near-spherical shape precipitates are mainly composed of Al and Cu, which might be attributed to the stable θ phase (Al_2_Cu) [[21], [22], [23]]. Whereas, as expected the large-size dispersoids are mainly composed of Mn and Cu [22]. As reported in the literature, most precipitates are identified as an alloy of Al–Cu. This is further explored through STEM-EDX elemental mapping conducted at a pixel size of 2.3 nm. However, this resolution likely posed challenges in correlating the intensity observed in EDS maps with the precipitates' images, which appear as white dots in the corresponding DF-STEM image in Fig. 3(a). In addition, the contrast observed in HADDF-STEM images for a large-size precipitate in Fig. 3(a) was found to be in line with the elemental composition of precipitates in the Al matrix. The contrast from the large-size dispersoids in Fig. 3 (a) is higher as compared to the Al matrix because they contained Cu and Mn elements than the Al matrix.

The Young's modulus ($Y_{M}$) map was generated next, from the same region was generated where STEM-EDS analysis was applied to determine the elemental composition. In this case, $Y_{M}$) map has been using STEM-EELS low-loss technique. Post-processing analysis of the acquired STEM-EELS datasets included the generation of thickness and $Y_{M}$ maps. The obtained results on thickness map from these datasets revealed that the specimen thickness was around 117 ± 6 nm which indicates a good value to main the strain perpendicular to the specimen planes. It is to be noted that as the specimen was tilted along [001]_Al_ zone axis, the bulk plasmons, being longitudinal oscillators in nature, will oscillate in the same [001]_Al_ direction. This also implies that the *Y*_*M*_ map will be projected along [001]_Al_ direction as well. From the acquired low-loss STEM-EELS spectrum image datasets, the value of E_p_ of the bulk plasmons was extracted and utilized to determine *Y*_*M*_ locally at each pixel point using equation (3). The analysis of STEM-EELS revealed an average value of E_p_ of nearly 14.9 eV for the Al matrix region which agrees with the literature. Whereas its average values for the small precipitates and dispersoids were found to be in the range of 15.1 eV and 15.2 eV, respectively. Subsequently, $Y_{M}$ was generated per equation (3) and the obtained results are presented in Fig. 3 (b). The color scale utilized in the image represents the range of $Y_{M}$ values which span from 71 to 79 GPa. This range encompasses the documented value of 73.1 GPa for the Al 2024 alloys [15]. The presented results further indicate that the Al matrix has a significantly different $Y_{M}$ in the precipitate regions from the matrix regions. It further implies that the presence of interatomic bonding among the Al atoms becomes stronger due to various reasons such as strain, composition, and dislocations at the interfacial regions of the precipitate-matrix. The Young's modulus map in Fig. 3(b) represents the spatial variation of the $Y_{M}$ values in the alloy at the nanoscale, which is generally taken as constant value while determining the uniaxial tensile or compression stresses of the alloys from the residual strains determined with diffraction-based techniques. In this way, the observed dynamic nature of the $Y_{M}$ at the nanoscale will surely be reflected on the corresponding residual stress maps whenever these are obtained from residual strain maps.

**Fig. 4:**
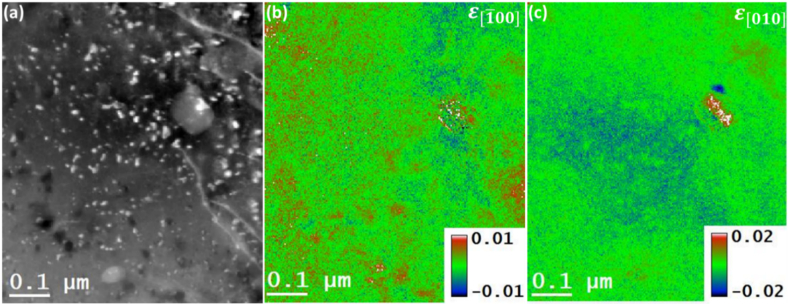
Strain analysis using 4D-STEM technique to the corresponding HAADF-STEM image. (a) HADDF-STEM image of the region of interest (b) the normal strain in the [$\bar{1}$ 00] direction, (c) the normal strain in the [010] direction.

As mentioned earlier, the 4DSTEM datasets were acquired from the same rectangular enclosed region in Fig. 1(a) to generate strain maps. The strain fields were computed numerically in STEMx by employing equation (2), which involves taking derivatives of the displacement. By utilizing this approach, the normal strain components $\varepsilon_{xx}$ and $\varepsilon_{yy}$ were generated along the [$\bar{1}$ 00] and [010] directions, respectively. The obtained results are presented in Fig. 4. Notice the strain field's color scale corresponds to the range from −0.02 % to 0.02 % in the [$\bar{1}$ 00] direction and from −0.01 % to 0.01 % in the [010] direction. This color-coded representation effectively distinguishes the nature of the strain, enabling clear visualization and interpretation of the material's deformation characteristics. In [$\bar{1}$ 00] direction, generally tensile strain was found to be dominating in the vicinity of large and small size precipitates, and it can be stated qualitatively that the larger part of the region was found in the state of tensile strain as compared to compressively strained regions. Whereas in the [010] direction, qualitatively speaking, the compressively strained regions were found to be in larger fractions than the regions under tensile strains. The presence of the alloying elements in the Al matrix explains the variations of the residual strains across the Al matrix. The larger strain fields in the vicinity of the precipitates compared to the Al matrix may be attributed to the coherent nature of precipitates at the underaged condition of the Al 2024 alloy, generating coherency strains. This ultimately plays a major role in controlling the movement of dislocations, which ultimately dictates the mechanical behavior of the Al2024 alloy [24].

**Fig. 5:**
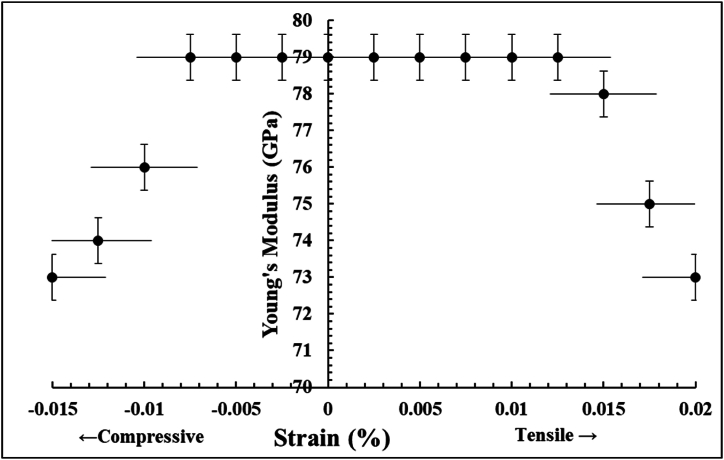
The relation between the Young's modulus and the residual strain extracted from $Y_{M}$ and strain maps.

Young's modulus is an intrinsic property governed by the interatomic forces between atoms [25] and, therefore, strongly depends on the strain. The values of $Y_{M}$ and strains were determined from the maps presented in Fig. 3 (b), and Fig. 4 (b), respectively. The obtained results are presented in Fig. 5. It can be noticed from the presented plot in Fig. 5 that for the tensile strains higher than 0.015 %, the value of $Y_{M}$ reaches a value of ∼ 73 GPa. In other words, $Y_{M}$ decreases about ∼ 7 % compared to its values for strains below 0.005 %. Whereas for compressive strains below −0.01 %, the $Y_{M}$ starts decreasing and reaches the same value of ∼ 73 GPa. It implies that the *Y*_*m*_ decreases ∼ 7 % for strain values above −0.005 %. The observed decrease in $Y_{M}$ beyond certain tensile and compressive domains indicates the weakening of Al–Al bonds in the alloy. This phenomenon can be elucidated using the Lennard-Jones model for the atoms in materials that undergo large displacements or transform to new bonding states either due to tensile or compressive strains [20]. In other words, for the regions under tensile strain, the atoms are drifted apart, and the interatomic potential diminishes, signifying a weakening of the bond and a consequent decrease in $Y_{M}$ value. Similarly, in the compressively strained region, the atoms approach closer to each other, and this results in the increase of repulsive forces, leading to a weakened bonding state and hence also leads to a reduction in $Y_{M}$. The observed decrease in $Y_{M}$ at a smaller value of compressive strains than tensile strains is also linked to the shape of Lennard-Jones potential as a function of strains or displacements. In other words, the Lennard-Jones potential is steeper for compressive strain than in the case of tensile strains. The deviation of *Y*_*m*_ values from its constant nature beyond certain strains will influence the residual stress maps. This result will very likely impact the applications where *Y*_*m*_ of the alloys is presumed to be constant [12,26,27].

**Fig. 6:**
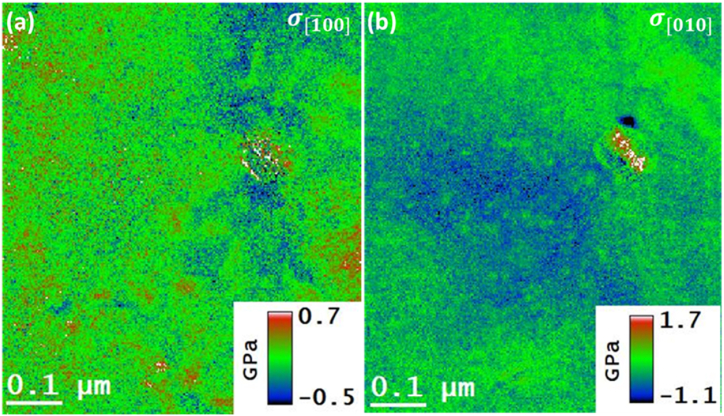
The corresponding residual stress maps are acquired by multiplying the $Y_{M}$ map and the residual strain maps. (a) normal stress in [$\bar{1}$ 00] direction, (c) normal stress in [010] direction.

The residual stress maps can be obtained by multiplying the *Y*_*m*_ map of Fig. 3 (b) with the residual strain maps of Fig. 4 (b) and Fig. 4 (c) in accordance with the Hooke's law i.e., equation (5). The determined normal stress maps in the [$\bar{1}$ 00] and [010] directions are presented in Fig. 6 (a), and Fig. 6 (b), respectively. The color scale in the presented residual stress maps spans from −0.5 to 0.7 GPa for the case of [$\bar{1}$ 00] direction and from −1.1 to 1.7 GPa for [010] direction. Both scales cover the value of the yield stress (i.e., 0.345 GPa) for the Al 2024 alloy [15]. By comparison of Fig. 4 (a) and 4(b), qualitatively speaking, the residual stress is more tensile at the precipitates in the [$\bar{1}$ 00] direction than in the [010] direction, while in the [010] direction, more region is under compressive stresses than the [$\bar{1}$ 00] direction. The presence of variations of residual stresses, especially at the precipitate-matrix interfaces, is attributed to the degree of coherency of those interfaces and the existence of alloying elements different from Al atoms [14].

The sensitivity and accuracy of the resultant residual strain maps are discussed next, and they depend on the 4DSTEM experimental conditions, such as the camera length, beam convergence angle, and the detector type used to record the diffraction patterns. The camera length set during data acquisition controls the field-of-view (FoV) of the diffraction patterns for the given area of the pixel size of the detector. It hence controls the accuracy of the obtained strain maps. In the present investigation, a camera length of 37 mm was selected so that the diffraction spots or disks could have been recorded on the FoV of the K3 camera. In this way, the pixel size in the diffraction patterns represented a strain resolution of 0.001, which is five times smaller than the elastic limit of the Al 2024 alloy. The beam convergence angle can also influence the accuracy of the obtained strain maps. A larger convergence angle means a smaller STEM probe size which produces larger diffraction spots or discs in the pattern patterns. This leads to the overlapping of the discs, which must be avoided. The detective quantum efficiency (DQE) of the detector used controls the speed of acquiring the 4DSTEM datasets. As expected, the direct electron detectors provide the speed advantage over their scintillator-based counterparts, but also the edges of the diffraction discs are sharper due to the higher modular transfer functions (MTF) of the former than the latter detectors. In summary, the optimized value of the cameral length (∼37 mm), the convergence angle (≈0.6mrad), and the direct electron detector type translated to less noise and high speed for the 4DSTEM datasets. In other words, it helped obtain strain distributions that were good quality and possessed high sensitivity.

The accuracy and sensitivity of the obtained $Y_{M}$ maps depend on the dispersion and detector types used to record the EELS spectra during the acquisition of STEM-EELS datasets. The value of the dispersion set for the recording of the low-loss EELS spectra controls the accuracy and sensitivity of measuring the energy of the bulk plasmon peak. In other words, the dispersion of the EELS spectra enables recording the changes in the energy of the plasmon related to the bonding, elemental composition, and strains. In this way, a higher dispersion enhances the ability to resolve fine energy differences, which can be correlated with changes in the material's mechanical properties. As mentioned earlier, a value of 0.09 eV/ch was set during EELS data acquisition that resulted in measuring *Y*_*m*_ with ∼1.5 % sensitivity and 0.5 % accuracy. The precision of measuring $Y_{M}$ also depends on the detector type used, again as expected, it can be improved using direct electron detectors due to their better DQEs and MTFs than the scintillator-based counterparts. The error or sensitivity of residual stresses was estimated in the following way. The energy dispersion (δE) or pixel value in the EELS spectrum used during the experiments represents the error or sensitivity of measuring the plasmon energy $\left(E_{p}\right)$. This is because a shift of 2 pixels will be real as per the Nyquist limit. The details on determining the error or sensitivity in Young's modulus are given below:

The error in plasmon energy determination $\left(\delta E_{p}\right)$ is given below:

(6) $$E_{p}^{\prime }=E_{p}+\delta E_{p}$$

The error or sensitivity Young's modulus $\left(\delta Y_{M}^{\prime }\right)$ is then can be represented as below:

(7) $$\delta Y_{M}^{\prime }=2.5\;\left(\frac{\delta E_{p}}{E_{p}}\right)Y_{M}$$

The $\delta Y_{M}^{\prime }$ in equation #2 equals to 1.5 % error in the Young's modulus of aluminum metal $\left(Y_{M}\right)$ for the dispersion of 90 meV.

In the same way, the error or sensitivity in strain quantification was estimated to a single pixel size ($\delta d=d^{\prime }-d_{0})$ of the CMOS detector in 4DSTEM datasets. This leads to the determination of strain error $\left(\delta \varepsilon^{\prime }\right)$ in the following way:

(8) $$\delta \varepsilon^{\prime }=\left(\frac{\delta d}{d_{0}}\;\frac{1}{\varepsilon }\right)\varepsilon$$

For (111) planes of aluminum and δd=0.008nm, the strain error $\left(\delta \varepsilon^{\prime }\right)$ was estimated to be nearly 3.5 %. As per Hooke's law, the residual stresses (σ) are given as the product of strain and Young's modulus. This means the error in residual stresses $\left(\delta \sigma^{\prime }\right)$ can be expressed in the errors of strain and Young's modulus in the following way:

(9) $$\frac{\delta \sigma^{\prime }}{\sigma }=\sqrt{{\left(\frac{\delta \varepsilon^{\prime }}{\varepsilon }\right)}^{2}+{\left(\frac{\delta Y_{M}^{\prime }}{Y_{M}}\right)}^{2}}$$

In this way, an error of 1.5 % in Young's modulus, and 3.5 % in residual strain result in an accumulated error or sensitivity of 3.9 % in residual stresses.

It is believed that the presented methodology of mapping the residual stresses is equally applicable to measuring stresses in both isotropic and anisotropic metal alloys, including those produced through additive manufacturing methods [28]. The scope and application of the 4DSTEM technique allow the mapping of residual strains without damaging the structure of the alloys. However, this can be achieved only by adopting appropriate electron beam currents and energies during the data acquisition. Consequently, this method might be categorized as " destructive”, depending on the degree of damage inflicted onto the sample by the electron beam. Hence depending upon the utilized electron beam conditions, the presented methodology can be considered destructive or nondestructive.

## 4 Conclusion

The correlated multimodal STEM-based analyses allow for determining the residual stresses in metal alloys at the nanoscale. The residual stress maps of Al2024 alloys can be realized by carrying out the mapping of the residual strain and *Y*_*m*_ with 4DSTEM and STEM-EELS techniques, respectively. The overall accuracy and sensitivity of the obtained results depend on the experimental conditions used during the 4DSTEM and STEM-EELS datasets, and the presented datasets possessed sensitivities of 0.5 % and 1.5 %, respectively. The presented *Y*_*m*_ vs. residual strain plot is critical to understanding the mechanical behavior of alloys as they can be used to determine threshold values of tensile and compressive strains at which *Y*_*m*_ starts changing. In conclusion, the presented methodology is a powerful way of determining residual stresses and strain-dependent *Y*_*m*_ of both isotropic and anisotropic metal alloys at the nanoscale.

## CRediT authorship contribution statement

**Mohamed E. Daoud:** Writing – original draft, Project administration, Data curation. **Inas Taha:** Methodology, Investigation. **Mohamed Helal:** Investigation. **H. Kamoutsi:** Investigation. **G.N. Haidemenopoulos:** Methodology, Investigation. **Kamran A. Khan:** Project administration, Methodology, Investigation. **Dalaver H. Anjum:** Writing – review & editing, Supervision, Funding acquisition, Conceptualization.

## Declaration of competing interest

The authors declare that they have no known competing financial interests or personal relationships that could have appeared to influence the work reported in this paper.

## References

[1] Tabatabaeian A., Ghasemi A.R., Shokrieh M.M., Marzbanrad B., Baraheni M., Fotouhi M. (2022). Residual stress in engineering materials: a review. Adv. Eng. Mater..

[2] Schajer G., Prime M., Withers P. (2022). Why is it so challenging to measure residual stresses?. Exp. Mech..

[3] Withers P. (2007). Residual stress and its role in failure. Rep. Prog. Phys..

[4] Sim W.-M. (2011). 3rd International Conference on Distorsions Engineering.

[5] Jiang X., Wei Y., Zhou J., Zhan K., Ding Z., Liang S.Y. (2023). Residual stress generation and evaluation in milling: a review. Int. J. Adv. Des. Manuf. Technol..

[6] Jiang G., Haiyang F., Bo P., Renke K. (2021). Recent progress of residual stress measurement methods: a review. Chin. J. Aeronaut..

[7] Meng Z., Tian C., Li P. (2018).

[8] Sutton M., Reynolds A., Wang D.-Q., Hubbard C. (2002). A study of residual stresses and microstructure in 2024-T3 aluminum friction stir butt welds. J. Eng. Mater. Technol..

[9] Sapienza S. (2021). Measurement of residual stress and Young's modulus on micromachined monocrystalline 3C-SiC layers grown on< 111> and< 100> silicon. Micromachines.

[10] Kandil F., Lord J., Fry A.T., Grant P. (2001).

[11] Zhu W., Marin E., Sugano N., Pezzotti G. (2017). Tensor-resolved Raman spectroscopic analysis of wear-induced residual stress fields in long-term alumina hip-joint retrievals. J. Mech. Behav. Biomed. Mater..

[12] Greco A., Sgambitterra E., Furgiuele F. (2021/10/01/2021). A new methodology for measuring residual stress using a modified Berkovich nano-indenter. Int. J. Mech. Sci..

[13] Salvati E., Romano-Brandt L., Mughal M.Z., Sebastiani M., Korsunsky A.M. (2019/04/01/2019). Generalised residual stress depth profiling at the nanoscale using focused ion beam milling. J. Mech. Phys. Solid..

[14] Kamoutsi H. (2021). Effect of precipitate coherency on the corrosion-induced hydrogen trapping in 2024 aluminum alloy. Int. J. Hydrogen Energy.

[15] Committee A.I.H. (1990). Metals handbook vol 2: properties and selection: nonferrous alloys and special-purpose materials. ASM Int.

[16] Howe J.M., Oleshko V.P. (2004). Application of valence electron energy-loss spectroscopy and plasmon energy mapping for determining material properties at the nanoscale. Microscopy.

[17] Gilman J.J. (1999). Plasmons at shock fronts. Phil. Mag. B.

[18] Oleshko V.P., Murayama M., Howe J.M. (2002). Use of plasmon spectroscopy to evaluate the mechanical properties of materials at the nanoscale. Microsc. Microanal..

[19] Hÿtch M., Snoeck E., Kilaas R. (1998). Quantitative measurement of displacement and strain fields from HREM micrographs. Ultramicroscopy.

[20] Starke E.A., Staley J.T. (1996). Application of modern aluminum alloys to aircraft. Prog. Aero. Sci..

[21] Cochard A. (2017). Natural aging on Al-Cu-Mg structural hardening alloys–Investigation of two historical duralumins for aeronautics. Mater. Sci. Eng., A.

[22] Xiao X., Zhou Z., Liu C., Cao L. (2022). Microstructure and its effect on the intergranular corrosion properties of 2024-T3 aluminum alloy. Crystals.

[23] Staszczyk A., Sawicki J., Adamczyk-Cieslak B. (2019). A study of second-phase precipitates and dispersoid particles in 2024 aluminum alloy after different aging treatments. Materials.

[24] Zhu C., Lv K., Chen B. (2020). On the S-phase precipitates in 2024 aluminum alloy: an atomic-scale investigation using high-angle annular dark-field scanning transmission electron microscopy. J. Mater. Res..

[25] Callister W.D., Rethwisch D.G. (2020).

[26] Salvati E., Romano-Brandt L., Mughal M., Sebastiani M., Korsunsky A. (2019). Generalised residual stress depth profiling at the nanoscale using focused ion beam milling. J. Mech. Phys. Solid..

[27] Sebastiani M. (2020). Nano-scale residual stress profiling in thin multilayer films with non-equibiaxial stress state. Nanomaterials.

[28] Fedorenko A. (2021). Anisotropy of mechanical properties and residual stress in additively manufactured 316l specimens. Materials.

